# Diagnostic Value of Labial Minor Salivary Gland Biopsy: Histological Findings of a Large Sicca Cohort and Clinical Associations

**DOI:** 10.3390/diagnostics13193117

**Published:** 2023-10-03

**Authors:** Konstantinos Triantafyllias, Mirjam Bach, Mike Otto, Andreas Schwarting

**Affiliations:** 1 Department of Rheumatology, Acute Rheumatology Center, 55543 Bad Kreuznach, Germany; 2 Department of Internal Medicine I, Division of Rheumatology and Clinical Immunology, University Medical Center, Johannes Gutenberg University, 55131 Mainz, Germany; laanmi@web.de; 3 Institute for Pathology, 54292 Trier, Germany; 4 Department of Rheumatology, Karl-Aschoff Clinic, 55543 Bad Kreuznach, Germany

**Keywords:** minor salivary gland biopsy, Sjogren’s syndrome, immunohistochemistry, sicca, focus score, chronic pain

## Abstract

(1) Background: The aim of this study was to analyze labial minor salivary gland biopsy (MSGB) findings of a large sicca cohort and to examine their associations with Sjogren’s syndrome (SS)-associated laboratory markers, phenotypic characteristics and systemic manifestations. Moreover, we sought to explore the ability of MSGB to identify SS patients among subjects with pre-diagnosed fibromyalgia (FM). (2) Methods: Included were all patients of three rheumatology departments having undergone a diagnostic MSGB within 9 years. Next to the examination of histological and immunohistochemical findings, we focused on activity and chronicity parameters of the underlying disease, autoantibodies, presence of systemic and hematologic involvement, as well as chronic pain and SS comorbidities. (3) Results: Among the 678 included patients, 306 (45.1%) had a positive focus score (FS). The remaining patients (*n* = 372) served as control subjects. There were significant correlations between FS and hypergammaglobulinemia (*p* < 0.001), ANA and rheumatoid factor positivity (both; *p* < 0.001), a weak significant correlation with erythrocyte sedimentation rate (rho = 0.235; *p* < 0.001) and a negative correlation with nicotine use (*p* = 0.002). Within the primary SS subgroup, FS was associated significantly with glandular enlargement (*p* = 0.007) and systemic hematologic manifestations (*p* = 0.002). Next to FS, CD20 cell staining showed an excellent diagnostic performance in the diagnosis of SS by an area under the curve of 0.822 (95%CI 0.780–0.864; *p* < 0.001). Interestingly, 42.1% of all patients with fibromyalgia (FM) having received an MSGB could be diagnosed with SS. (4) Conclusion: By examining one of the largest cohorts in the literature, we could show that MSGB histological and immunohistochemical findings not only play a key role in the classification and diagnosis of SS but could also provide important information regarding SS phenotype and systemic manifestations. Furthermore, MSGB may help differentiate patients with FM from patients with subclinical SS who suffer primarily from chronic pain.

## 1. Introduction

Sjogren’s syndrome (SS) is one of the most common autoimmune diseases, characterized primarily by inflammatory affection of the secretory glands [1,2]. In addition to glandular manifestations, patients often suffer from symptoms severely affecting quality of life, such as fatigue, chronic musculoskeletal pain, joint swelling and stiffness [3,4]. Moreover, in up to 70% of patients, systemic organ involvement can occur, affecting, among others, the lungs, central and peripheral nervous system, kidneys, skin and gastrointestinal tract [4,5]. Hematologic manifestations can also be observed in the context of SS, and approximately 5% of SS patients develop a malignant lymphoma during the course of the disease [6,7].

The classification and diagnosis of SS require a full clinical, laboratory and paraclinical evaluation, as well as the performance of specific ophthalmological and dental tests, such as the evaluation of the ocular surface staining score or the van Bijsterveld score, the unstimulated whole saliva flow rate and Schirmer’s test [8]. Moreover, two of the most crucial items of the current (2016) ACR/EULAR classification criteria of SS are SSA-Ro antibodies and histopathological results of labial minor salivary gland biopsy (MSGB). Particularly, SSA-Ro positivity and the finding of focal lymphocytic sialadenitis (focus score (FS) ≥ 1/4 mm^2^) each correspond to 3 scoring points out of 4 needed to reach a positive SS classification [8]. Interestingly, MSGB was one of the most important items in all previous classification criteria of SS [9,10].

The value of MSGB regarding classification and diagnosis of SS has been well established over the years. Moreover, several studies have found a high predictive performance regarding hematologic involvement, as well as associations of MSGB with various clinical disease characteristics. For instance, Chatzis et al. [11] and Risselada et al. [12] showed a high prognostic value of MSGB in the diagnosis of lymphoma, with high FS patients being at high risk for this hematologic manifestation. Furthermore, in the study of Kakugawa et al., higher labial gland biopsy focus scores (≥4) were risk factors for airway diseases during a retrospective examination of 101 SS patients [13]. On the other hand, La Rocca et al. reported that SS patients with intestinal lung disease had less pronounced sialadenitis structural changes [14].

However, data on the diagnostic value of additional immunohistochemical markers, such as CD3, CD20, CD21 and IgG4 staining, are scarce and/or based on small cohorts (for instance: *n* = 45 [15], *n* = 37 [16] and *n* = 50 [17]). Moreover, the vast majority of larger cohorts originate from registries rather than clinical trials. For instance, Daniels et al. analyzed registry data and showed that patients with high FS had higher rates of SSA-Ro antibodies, rheumatoid factor (RF), antinuclear antibodies (ANA) and hypergammaglobulinemia [18]. A positive relationship between hypergammaglobulinaemia and FS could also be shown by Brennan et al. during the investigation of risk factors for positive MSGB results in a “dry mouth” cohort [19]. Moreover, higher FS was associated with abnormal tear production in a retrospective study of 141 patients [20]. Interestingly, Fei et al. did not find an association between systemic involvement and FS in a cohort of 77 patients [21]. However, one should keep in mind that most of these studies were performed using older SS classification criteria, evaluated only FS (and not immunohistochemical markers), and included a low number of patients and/or cohorts with low rates of systemic organ involvement. 

Another important point of discussion in patients with SS is a common overlap with chronic pain syndromes [22,23]. Registry data report the presence of fibromyalgia (FM) in 14% to 55% of patients with primary SS (pSS) [24,25,26]. Furthermore, various SS-associated antibodies have been found in FM patients, pointing to a possible link between these two syndromes [27]. Nevertheless, biopsy-based data regarding this association are scarce. Additionally, it is unknown which percentage of patients with FM and other chronic conditions suffer from SS with a subclinical or mildly symptomatic SS disease course.

Thus, the primary aim of this study was to examine the value of MSGB in the diagnosis of SS focusing not solely on the focus score but also on the value of additional immunochemistry examinations in one of the largest MSGB cohorts ever examined. Secondarily, we aimed to evaluate whether MSGB can be helpful in differentiating patients with FM and other chronic pain disorders from chronic pain sufferers with subclinical forms of SS.

## 2. Materials and Methods

**Figure 1:**
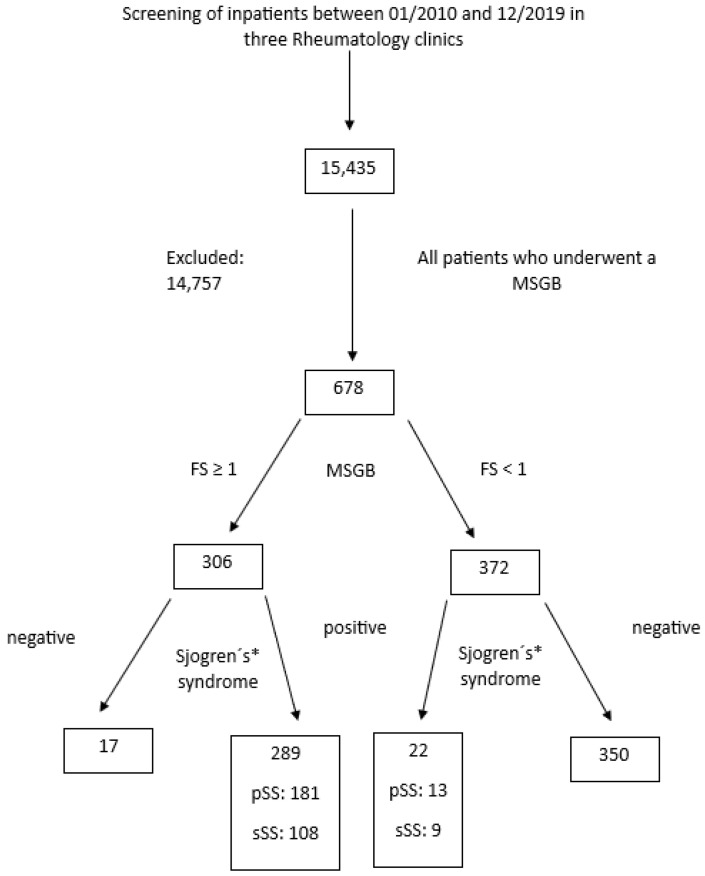
Flowchart describing included and excluded cases and controls of the study population. MSGB: minor salivary gland biopsy; FS: focus score. * Sjogren’s: ACR/EULAR classification.

Study population: Recruited in this study were all inpatients from three large rheumatological centers in Germany who had undergone a diagnostic MSGB between January 2010 and December 2019. The diagnostic MSGB was performed in the case of clinical suspicion for the presence of sicca symptoms (SS) and an accompanying abnormal Schirmer’s or Saxon’s test and/or a positive SS-associated autoantibody, such as SSA-Ro and/or SSB-La. Patients with sole SSB-La positivity who did not fulfill SS classification criteria [8] were included in the control group. Excluded from the study were patients with a history of head and neck radiation treatment, active hepatitis C infection, acquired immunodeficiency syndrome, sarcoidosis, amyloidosis, graft versus host disease, IgG4-related disease [8] and an age of ≤18 years. FM was established according to the American College of Rheumatology (ACR) diagnostic criteria by Wolfe et al. [28]. In the flow chart of Figure 1, we present the screening procedure and present all data regarding included and excluded cases and controls. 

Study data were collected partly in a prospective and partly in a retrospective manner. The study was reviewed and approved by the local standing committee for ethical conduct in adherence to the Helsinki Declaration, and written informed consent was obtained from the study subjects.

**Figure 2:**
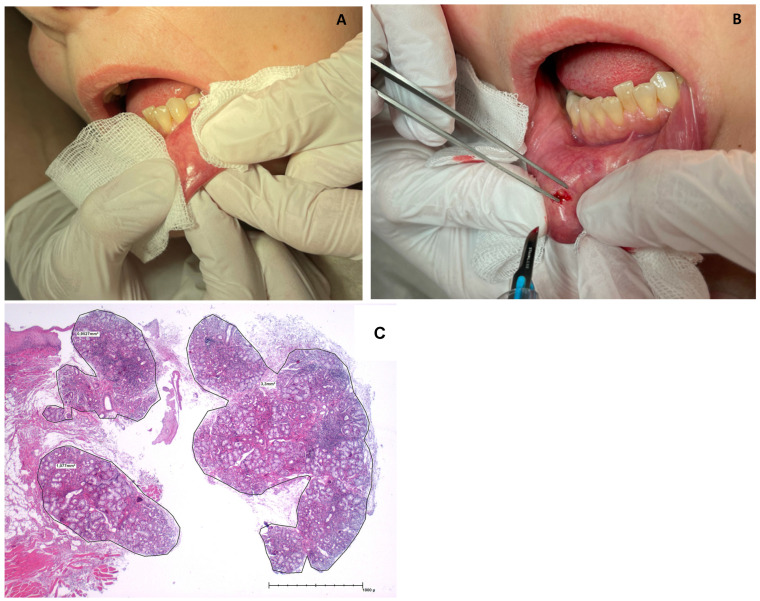
(**a**) Identification of a labial minor salivary gland. (**b**) Performance of minor salivary gland biopsy in a minimally invasive manner. (**c**) Hematoxylin and eosin (H&E) staining of salivary gland tissue with the finding of focal lymphocytic sialadenitis by a focus score of 6.0 in the context of primary Sjogren’s syndrome.

Minor salivary gland biopsy (MSGB) was performed based on the procedure described by Daniels et al. [29]. The lower lip was everted by a medical assistant, and by putting it under tension, the inner surface was exposed, and the minor salivary glands were identified (also through visible saliva excretion) (Figure 2). A local anesthetic was applied topically to the chosen spot(s), followed by small incision(s) mostly parallel to the vermilion border [30]. In the majority of the cases, we obtained at least 2 salivary gland biopsy items, as suggested by Fisher et al. [31], and the median surface of selected biopsies was ≥6.4 cm^2^.

The analyses of the biopsies were performed by experienced pathologists of a cooperating pathology institute (Pathology Medical Care Center, Trier, Germany) according to the at the time valid guidelines. Previous to 2016 (2010–2016), MSGB was assessed in accordance with the histopathologic criteria suggested by the European Study Group on Classification Criteria for Sjogren’s Syndrome in 2002 [32]. In 2017, new criteria were introduced by the “EULAR Sjogren’s syndrome study group”, and for that reason, all MSGB performed from 2017 and onwards were assessed by the new guidelines [31]. These included additional immunohistochemical assessments of CD3, CD20, CD21 and IgG4-positive cells [15,33]. Subsequently, sections were processed using a Roche BenchMark Ultra tissue staining system with the Roche ultraView Universal DAB Detection Kit, based on a horseradish peroxidase/diaminobenzidine-chromogen antibody detection system. As primary antibodies, we used CD20 antibody (L26, Ventana), CD3 antibody (2GV6, Ventana), CD21 antibody (2G9, Cell Marque), and IgG4 antibody (MRQ-44, Cell Marque) in standard dilution (ready to use) from Ventana/Roche. 

FS was calculated in all patients by dividing the amount of all foci (aggregates with more than 50 mononuclear cells) by the surface of the gland and multiplying it by 4 to obtain the number of foci per 4 mm^2^ [31]. 

Data collection: Next to the examination of histological results, we focused on activity and chronicity parameters of the underlying disease, autoantibodies, and presence of systemic organ involvement, as well as on current and previous comorbidities. ILD screening was performed via pulmonary function tests and X-ray examinations of the chest in two planes. When pathologic results were found and/or when the patient presented typical symptoms/signs of ILD (i.e., cough, dyspnea), we additionally performed a high-resolution CT scan. Presence of inflammatory arthritis (joint swelling/tenderness) was evaluated clinically, and Raynaud’s phenomenon was assessed via patient history and/or by a cold test. 

Laboratory examinations included C-reactive protein (CRP), erythrocyte sedimentation rate (ESR), differential blood counts, renal parameters, immunoglobulin G (IgG) and immunoserological markers, such as ANA (detected on the Hep-2 cells by indirect immunofluorescence), RF, double-stranded DNA (ds-DNA) and ENAs (SSA-Ro, SSB-La, Sm, Scl-70 and U1-RNP, assessed by ELISA).

As part of the classification criteria for SS, patients also underwent a Schirmer’s test, which was considered abnormal if <5 mm after 5 min. Moreover, we examined saliva excretion by Saxon’s test, which is not part of the actual classification criteria but is still part of clinical practice and is still performed in many rheumatology centers, including ours (abnormal if <2.5 g after 2 min) [34,35]. We also took into account and documented the subjective experience of sicca symptoms (oral, ocular and other mucous membranes). Data regarding systemic organ involvement were grouped into different categories (lymphatic and hematologic system, glandular, cutaneous, renal, pulmonary, peripheral and central nervous system). We defined lymphatic abnormalities as clinical and/or sonographic diagnosed lymph node enlargement. Hematologic abnormalities were defined as the presence of leucopenia, anemia, thrombocytopenia, monoclonal gammopathy and/or the confirmed diagnosis of a hematologic disease, such as Non-Hodgkin- or Hodgkin-lymphoma. Glandular enlargement was defined as the presence of a visible and/or palpable enlarged submandibular and/or parotid glands. Moreover, we documented the presence of further rheumatological and other autoimmune diseases, such as inflammatory bowel diseases, Hashimoto’s thyroiditis, Basedow’s, vitiligo, autoimmune hepatitis and gastritis, multiple sclerosis and myasthenia gravis. Additionally, we registered the current medication of the patients, especially conventional synthetic and biologic disease-modifying antirheumatic drugs (DMARDs) and glucocorticoids (Table 1).

Statistical analysis: The assumption of normality of distribution was examined by the Saphiro–Wilk numerical test and quantile–quantile plots. In this work, continuous variables are presented as mean (S.D.) in the case of normal distributions and as median (25th/75th percentiles) when skewed. Categorical variables are presented as absolute (*n*) and relative (%) frequencies. For the comparison of categorical variables, we used the chi-squared test. Diagnostic performances of autoantibodies, subjective sicca symptoms, Schirmer’s test and Saxon’s test compared to the FS (reference) were examined by receiver operating characteristics (ROC). We also used ROC to evaluate the added value of immunohistochemical markers, such as CD3, CD20, CD21 and IgG4, in the diagnosis of SS. In order to examine differences between the characteristics of SS patients and control subjects, the *t*-test was used in the case of normally distributed variables and the Mann–Whitney U in the case of skewed distributions test or ANOVA in the case of more than two characteristics. To examine correlations between MSGB markers and continuous characteristics, Spearman’s (rho) or Pearson’s (r) correlation coefficients were used. All statistical calculations were performed by using IBM SPSSVR 23.0 software (Armonk, NY, USA).

## 3. Results

In total, 678 patients were included. Of the patients, 615 were females, the average age was 54.76 years, and the vast majority came from the broader southwest Germany district. Moreover, 306 patients (45.1%) had a positive FS. The remaining 372 patients (54.9%) with a negative FS served as a control group (rheumatoid arthritis, 14.9%; undifferentiated connective tissue disease, 19.3%; SLE, 4.5%; systemic sclerosis, 3.6%; psoriatic arthritis, 2.8%; polymyalgia rheumatica, 1.7%; overlap syndromes, 2.8%; further diseases, 10.1%). Descriptive characteristics of patients with primary and secondary SS and their statistical differences are shown in Table 1. Respective descriptive characteristics of patients with positive and negative FS and their statistical differences are shown in Table 2 and Table 3 for the whole population and the pSS, respectively.

### 3.1. Receiver Operating Characteristics (ROC)

**Figure 3:**
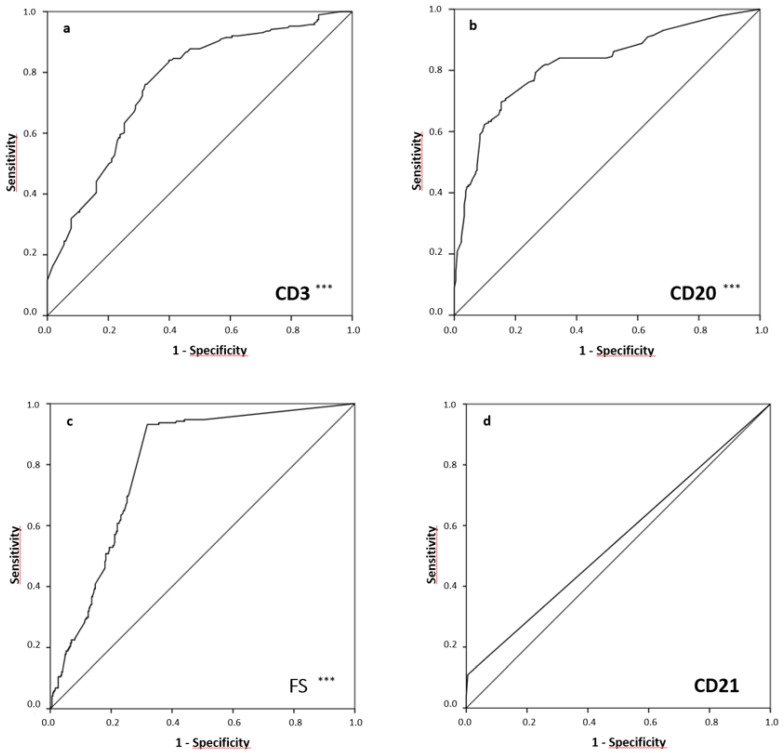
Receiver operating characteristics SS diagnosis vs. histology/immunohistochemistry data (**a**–**d**): (**a**) CD3 (AUC (95%-CI) = 0.761 (0.715, 0.808), *p* < 0.001), (**b**) CD20 (AUC (95%-CI) = 0.822 (0.780, 0.864), *p* < 0.001), (**c**) FS (AUC (95%-CI) = 0.797 (0.759, 0.835), *p* < 0.001), (**d**) CD21 (AUC (95%-CI) = 0.552 (0.495, 0.610), *p* = 0.072). SS: Sjögren’s syndrome; AUC: Area under the curve; 95%-CI: 95%-confidence interval; *p*: significance; FS: focus score; *** *p* < 0.001.

Diagnostic performances of FS, CD3, CD20 and CD21 (*n* = 420) regarding the presence of SS were evaluated via ROC (Figure 3). FS showed an excellent value in the diagnosis of SS by an AUC of 0.797 (95%CI, 0.759–0.835; *p* < 0.001). Interestingly, additional CD20-positive cell staining was associated with a further improved diagnostic performance by an AUC of 0.822 (95%CI, 0.780–0.864; *p* < 0.001). Moreover, CD3-positive staining was found to be of high diagnostic value, even though this was lower than the ones of CD20 and FS (AUC 0.761 (95%CI, 0.715, 0.808; *p* < 0.001)). On the contrary, CD21 and IgG4 cell staining did not show statistically significant results (AUC 0.552 (95%CI, 0.495, 0.610; *p* = 0.072) and AUC 0.534 (95%CI, 0.473, 0.595; *p* = 0.274), respectively). 

**Figure 4:**
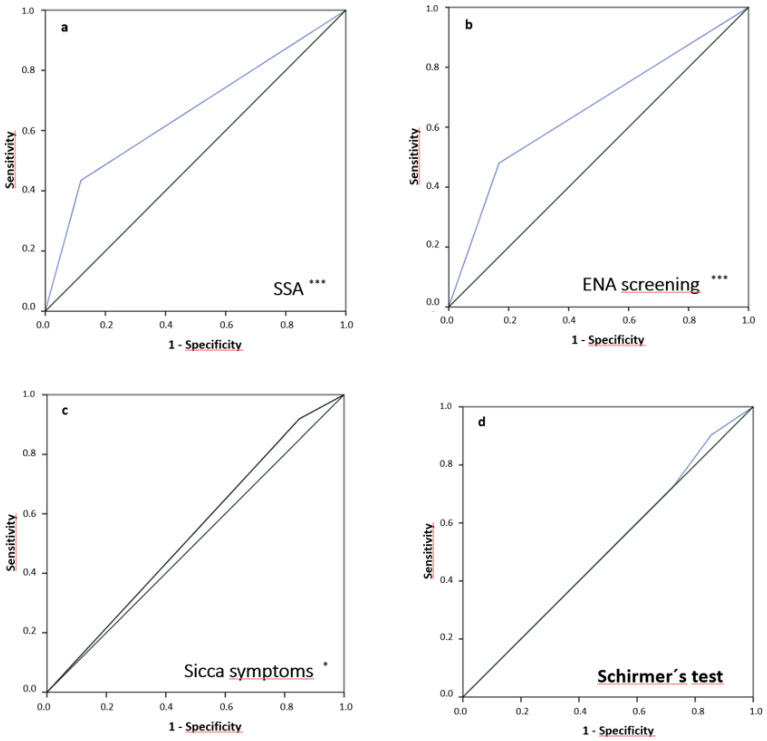
Receiver operating characteristics (focus score vs. further diagnostic items (**a**–**d**)): (**a**) SSA antibodies (AUC (95%-CI) = 0.658 (0.613, 0.703), *p* < 0.001), (**b**) ENA screening (AUC (95%-CI) = 0.656 (0.612, 0.700), *p* < 0.001), (**c**) subjective sicca symptoms (AUC (95%-CI) = 0.535 (0.489, 0.580), *p* = 0.031), (**d**) Schirmer’s test (AUC (95%-CI) = 0.507 (0.450, 0.565), *p* = 0.574). AUC: Area under the curve; 95%-CI: 95%-confidence interval; *p*: significance. (green line: no significant/predictive value). *, *** Significant differences between the two groups.

ROC was also performed within the whole group (*n* = 678) in order to test the diagnostic performance of SSA-, SSB antibodies, subjective sicca symptoms, Schirmer’s test and Saxon’s test in comparison to FS, which was taken as a reference. In these analyses, SSA antibodies showed the best diagnostic performance compared to FS by an area under the curve (AUC) of 0.658 (95% CI: 0.613–0.703) (Table 4, Figure 4).

### 3.2. Whole Population

In the whole population, level of FS also correlated significantly with the presence of hypergammaglobulinemia [1.27 (0.71–3.36, IQR) vs. 0.73 (0.0–1.76, IQR); *p* < 0.001], RF [1.51 (0.47–3.09, IQR) vs. 0.63 (0.0–1.31, IQR), *p* < 0.001] and with ANA positivity (rho = 0.280, *p* < 0.001). Moreover, subgroup analyses of the ANA patterns showed a statistically significant difference in the FS among different patterns (*p* < 0.001), with the highest median FS being seen in patients with a homogeneous pattern [1.42 (0.64–3.0, IQR)], compared to [0.33 (0.0–1.58, IQR)] in patients with a nucleolar pattern. Moreover, FS levels correlated weakly significantly with erythrocyte sedimentation rate (rho = 0.235, *p* < 0.001) but not with CRP (rho = −0.023, *p* = 0.6) (Table 5). 

Regarding the markers assessed by immunohistochemistry, significant strong correlations could be observed between the level of FS and the level of CD3-, and C20 cells detected in the biopsy specimens (rho = 0.659, *p* < 0.001 and rho = 0.692, *p* < 0.001, respectively) (Table 6). CD21-positive cells showed a moderate significant correlation with FS (rho = 0.292, *p* < 0.001), while the association of IgG4-positive cells with FS was poor (rho = 0.118, *p* = 0.014, respectively). Further associations of immunohistochemical markers (CD3, CD20, CD21, IgG4) with clinical and laboratory patient characteristics are presented in Table 6.

**Figure 5:**
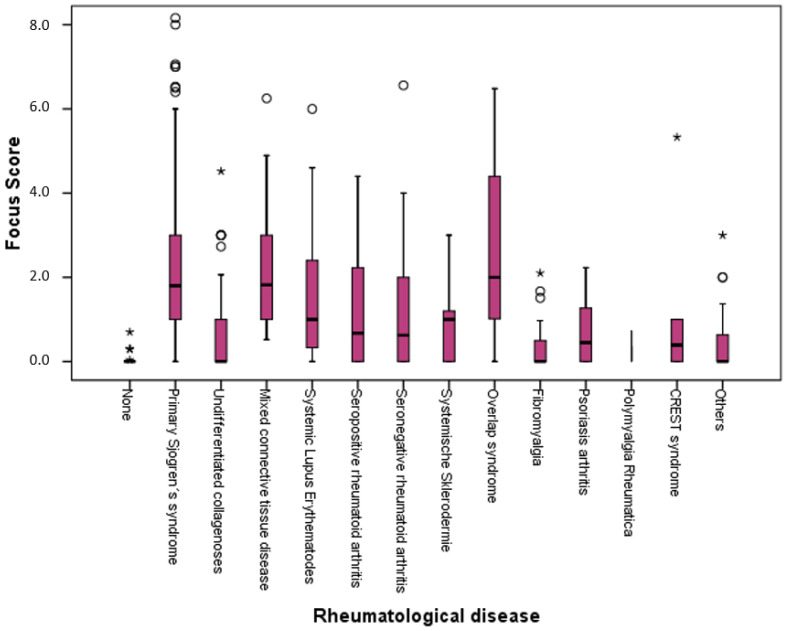
Comparison of the average value of focus score in patients with all included rheumatic diseases. pSS significantly higher compared to undifferentiated connective tissue disease, systemic lupus erythematosus, seropositive and seronegative rheumatoid arthritis, systemic sclerosis, fibromyalgia, psoriatic arthritis, polymyalgia rheumatica, CREST syndrome and other diseases (*p* < 0.001) but not significantly higher compared to mixed connective tissue disease and overlap syndromes (both; *p* > 0.05). pSS: primary Sjogren’s syndrome; *p*: significance, °: outlier, *: extreme value.

Regarding cardiovascular risk factors, the most significant correlation was found for nicotine use (negative association between the level of FS and nicotine; *p* = 0.002, Table 6). Depending on the underlying rheumatological disease, there were significant differences in FS levels (Figure 5). Diseases with the highest FS levels were pSS, mixed connective tissue disease and overlap syndromes. On the other hand, there was no significant difference between pSS and secondary SS (sSS) [1.8 (1.0–3.0, IQR) vs. 2.0 (1.06–3.19, IQR), *p* = 0.232]. Patients under DMARD had statistically significantly higher FS values compared to patients without [1 (0–2.34, IQR) vs. 0.87 (0–1.8, IQR); *p* = 0.004], but there were no significant differences regarding FS levels and glucocorticoid therapy.

### 3.3. Primary SS Subgroup

**Figure 6:**
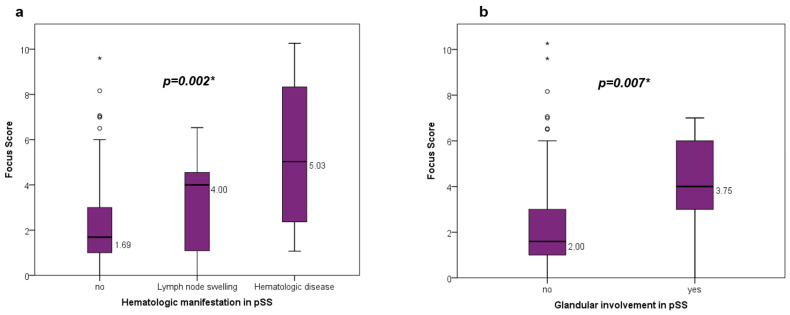
FocussScore comparisons in patients with pSS regarding (**a**) hematologic manifestations [4 (1–5, IQR) vs. 5.03 (1.72–9.3, IQR) vs. 1.69 (1–3, IQR); *p* = 0.002]; (**b**) glandular involvement [4 (2.98–6.2, IQR) vs. 1.6 (1–3, IQR); *p* = 0.007]. pSS: primary Sjogren’s syndrome; IQR: interquartile rate; *p* < 0.05, °: outlier; * *p* < 0.05.

Within the group of patients with pSS, there were significant correlations between the level of FS and the presence of glandular enlargement and hematologic manifestations (Table 6, Figure 6). ANA fine granular and centromere patterns were found to be associated with higher FS values. There were no significant correlations between FS and involvement of the solid examined organ systems (cutaneous, renal, pulmonary, nervous system; all; *p* > 0.05). Systemic involvement in the context of the disease correlated however significantly with ESR (*p* = 0.043).

### 3.4. MSGB and Fibromyalgia

Examining the frequency of the diagnosis of fibromyalgia (FM) in patients included in this study, we found that among the 159 patients who were admitted to the centers due to the diagnosis of primary fibromyalgia and received an MSGB, 63 (39.6%) had a positive FS. Moreover, 67/159 patients in total (42.1%) could be diagnosed with SS on the basis of the ACR-EULAR classification criteria (50 (31.4%) with pSS, 17 (10.7%) with sSS; Table 7). 

## 4. Discussion

In this study, we were able to show that MSGB findings not only play a key role in the classification and diagnosis of SS but could also provide important information regarding the presence of systemic/hematologic involvement in the context of the disease. Furthermore, our findings indicate that additional immunohistochemical staining with CD20- and CD3-positive cells can improve diagnostic MSGB value and that labial gland biopsies can help differentiate patients with FM from patients with subclinical SS who suffer primarily from chronic pain.

To our knowledge, this is one of the largest studies examining histological and immunohistochemical findings in a sicca cohort, including patients with both pSS and sSS. In particular, literature data on immunohistochemical staining are scarce, and the current findings may contribute to an increase in the diagnostic MSGB value. An additional advantage of this work is the fact that a further subgroup consisting of sicca patients with negative MSGB findings could be included and serve as an intrinsic control group. Furthermore, this study is one of the few to have examined relationships of histological findings with clinical, laboratory and also (not well-examined) SS patient-associated characteristics, such as chronic pain.

In our study, FS showed, as expected, excellent diagnostic performance in the diagnosis of SS. Interestingly, CD20-positive staining showed an even higher diagnostic value, pointing to an additional utility of this immunohistochemical marker. Moreover, CD3 and CD21 were also found to perform in a statistically significant manner and were associated with clinical and laboratory disease-associated parameters. These findings can be explained by the fact that CD20 and CD3 stainings can be helpful in identifying germinal centers (GCs) and in assessing the B/T cell ratio in foci [31]. Staining with CD21 can furthermore improve the identification of follicular dendritic cells, and even though isolated CD21 long isoforms cannot sufficiently confirm the presence of GCs, the combined presence of FDC and B/T cells can [31]. Similar to our study, Trivedi et al. were able to show that additional CD3, CD20 and CD45 staining had increased diagnostic certainty in a small cohort of 35 SS patients [15]. The higher diagnostic accuracy was explained through an improvement in the identification of lymphocytic infiltrates, particularly in cases of small lymphocytic clusters, which may be hard to appreciate on H&E within the salivary acini and ducts [15]. However, this study included a low count of patients, and the exact associations of these immunohistochemical markers with disease-associated characteristics were not examined.

In our exploration, MSGB was also taken as a reference to examine the diagnostic performance of other SS screening tools used in routine clinical practice. Regarding evaluated autoantibodies, the best diagnostic performance in comparison to FS could be shown for SSA-Ro, followed by SSB-La. This finding has been also supported by the working group having proposed the actual ACR/EULAR classification criteria, stating that the diagnostic role of SSA antibodies is higher than other SS-related antibodies [8]. For this exact reason, actual SS classification criteria have included solely SSA autoantibodies and not SSB-La, ANA or RF.

Interestingly, neither Schirmer’s nor Saxon’s tests were statistically significantly different between patient and control groups, pointing to low diagnostic performance in both detecting SS patients and differentiating non-specific sicca symptoms from SS-associated ones, as can be also seen by the results of the ROC analyses. The low specificity of tests such as Schirmer’s has also been pointed out by Chiu et al. in a single-center retrospective case–control study examining a large cohort (*n* = 505 patients) [36]. Moreover, van Nimwegen et al. showed poor diagnostic accuracy in a study that had as an objective the validation of the actual ACR/EULAR classification criteria [37]. On the other hand, such clinical tests have proven important in providing information regarding the severity of eye and mouth dryness. For these reasons, they are an important part of the actual ACR/EULAR classification criteria. Moreover, two further studies showed that Schirmer’s test results can correlate with FS [20,38].

In our study, FS showed a positive association with ANA and RF in the whole group, as well as in the pSS subgroup. Interestingly enough, a homogenous ANA pattern was associated with higher levels of FS in comparison to other patterns when examining our general cohort. However, when evaluating the pSS subgroup, fine-granular and centromere patterns were found to be associated with higher FS values. Similarly, Damoiseaux et al. found the fine-granular ANA pattern to be the most common in patients with SS [39]. Given the fact that SSA-Ro antibodies are the most prominent antibodies in pSS, these results are absolutely plausible. 

Since in our pSS cohort ESR was correlated with the level of FS, as well as with the presence of systemic involvement, FS may be seen as a possible marker for SS disease activity with a predictive value regarding the presence of a more severe disease. This has been also pointed out by Gu et al., who showed that higher FS could indicate a higher prevalence of systemic involvement in the context of the disease [40].

Interestingly enough, in the current study, not only disease but also patient-associated characteristics were found to be associated with FS. One of these factors was nicotine consumption: in the general cohort, patients who were smoking at the time of the MSGB had statistically lower FS levels compared to non-smokers. This finding can at first glance seem surprising because of the known detrimental effects of nicotine and a documented association with higher disease activity. It has, however, also been described in further studies and has been explained by a lower concentration of lymphocytes in the examined glands of smokers [41,42]. 

There were no significant differences in the level of FS between pSS and sSS. The highest FS values could be observed in patients with rheumatologic overlap syndromes, indicating a more aggressive disease course in these patients. An overall different phenotypic appearance of these patients with more severe sialadenitis, and thus the need for a different treatment strategy, should be further discussed. The examination of overlap syndromes can be nevertheless complicated due to a missing uniform definition and highly variable clinical pictures [43,44]. The same applies to the case of sSS since there are no separate specific classification criteria for this entity [8,45]. Moutsopoulos et al. did not find a difference in histopathology, RF or immunoglobulins between SS patients with and without additional rheumatoid arthritis [46]. Furthermore, Manoussakis et al. did not find significant FS differences between patients with pSS and sSS in the context of systemic lupus erythematosus [47]. 

Current disease-modifying antirheumatic drugs (DMARDs) showed a significant association with the level of FS in our study. Patients receiving DMARDs had statistically higher levels of FS, pointing to a more prominent use of these immunosuppressive medications in severe/aggressive disease courses. Interestingly enough, biologic DMARDs have been shown to reduce FS in different studies. For instance, treatment with abatacept resulted in a reduction of foci [48], and a 120-week-long therapy with rituximab resulted in a reduction of glandular infiltration in pSS cohorts [49]. 

In our cohort, only a minority of patients had present germinal centers in the MSGB (0.005%). Non-Hodgkin lymphomas and other hematologic conditions, such as monoclonal gammopathies, were also documented in few patients. Interestingly, these few patients showed statistically significantly higher FS in comparison to their counterparts. Based on these data, FS can prove to be a good prognostic marker for the occurrence of such additional complications and should be taken into account when screening patients for hematologic diseases [6,7,50]. Similar results could be shown by Chatzis et al., who found that patients with FS ≥ 4 developed lymphomas significantly faster than patients with FS < 4 [11].

Another important finding of this study was the high prevalence of underdiagnosed SS in patients with suspected chronic pain disorders, such as FM. This indicates the importance of MSGB in assisting SS classification and avoiding misdiagnosis. Even if a relationship between SS and FM has been described, no accurate data regarding the prevalence of undiagnosed SS among chronic pain sufferers are known. However, a nationwide Taiwanese study found that patients with FM had a hazard ratio of 2.0 to develop SS compared to control subjects [51]. Moreover, Torrente-Segarra et al. showed that patients with FM and SS had more intense somatic symptoms and subjectively higher disease activity [26].

Our study has some limitations. Firstly, one arm of the study was analyzed retrospectively. In this arm, we also included patients who had received an MSGB between the years 2010 and 2016. Even though FS interpretation has not radically changed over the years, data on more specific markers of MSGB, such as immunochemistry markers (CD3+, CD20+ cells, germinal centers), have been documented by our cooperating pathologists only during the prospective arm of the study, meaning after 2016. Secondly, since study data have been collected over several years, three different pathologists were involved in the analysis of the MSGB findings, which could have led to interobserver variability. Thirdly, we could not examine all items of the actual ACR/EULAR classification criteria, such as unstimulated whole saliva rate and ocular staining scores, due to the fact that these examinations are usually not performed in routine rheumatology practice and also not in our center. However, we included Saxon’s test and Schirmer’s tests in our analyses in order to include markers of both mouth and eye dryness. Furthermore, since we did not perform examinations of cognitive function via a specific mental test in every included patient, we did not include data on cognitive impairment in our study.

To conclude, by examining one of the largest cohorts in the literature, we showed that specific immunohistochemistry examinations can increase the diagnostic performance of MSGB. Moreover, MSGB findings do not only play a key role in the classification and diagnosis of SS but could also provide important information regarding the presence of systemic hematologic and glandular involvement. At last, MSGB may help in differentiating patients with FM and other chronic pain disorders from patients with subclinical SS who suffer primarily from chronic pain. The results of these explorations should be controlled and validated in further studies. 

## Figures and Tables

**Table 1:** Descriptive patient characteristics (primary Sjogren’s syndrome subgroup (pSS) and secondary Sjogren’s syndrome subgroup (sSS)).

	pSS (*n* = 194)	sSS (*n* = 117)	Significance (*p*)
Age † (years)	56.75 ± 13.07	57.18 ± 12.86	0.771
Gender (female)	189 (91.3%)	110 (89.4%)	0.626
Arterial hypertension (yes)	70 (33.8%)	48 (39%)	0.552
Type 2 diabetes (yes)	11 (5.3%)	10 (8.1%)	0.385
Hyperlipidemia (yes)	58 (28.1%)	32 (26.0%)	0.998
Nicotine use (smokers)	34 (16.4%)	20 (16.3%)	0.912
HDL † (mg/dL)	63.14 ± 18.33	65.56 ± 17.83	0.387
LDL † (mg/dL)	131.54 ± 44.52	123.42 ± 35.12	0.176
Total cholesterol † (mg/dL)	202.46 ± 47.47	196.57 ± 40.71	0.345
Systolic blood pressure † (mmHg)	121.13 ± 16.65	120.93 ± 15.32	0.954
Diastolic blood pressure † (mmHg)	74.77 ± 14.10	74.19 ± 8.72	0.808
Undifferentiated connective tissue disease		15 (12.2%)	
Mixed connective tissue disease		11 (8.9%)	
Systemic lupus erythematosus		21 (17.1%)	
Seropositive rheumatoid arthritis		16 (13%)	
Seronegative rheumatoid arthritis		15 (12.2%)	
Systemic sclerosis		6 (4.9%)	
Overlap syndrome		20 (16.3%)	
Psoriatic arthritis		5 (4.1%)	
CREST syndrome		5 (4.1%)	
others		9 (7.3%)	
Focus Score ‡	1.80 (1–3)	2.00 (1.06–3.19)	0.232
Gland area ‡ (mm^2^)	6.90 (3.80–10.02)	6.29 (3.35–8.68)	0.213
Number of samples ‡	2 (1–2)	2 (1–2)	0.504
CD3 ‡	74.50 (52.25–150)	80.00 (53.75–200)	0.636
CD20 ‡	50.00 (20–100)	51.00 (16.25–50)	0.810
ENA screen (positive)	90 (43.5%)	68 (55.3%)	0.111
SSA antibodies (positive)	79 (38.2%)	62 (50.4%)	0.103
SSB antibodies (positive)	25 (12.1%)	17 (13.8%)	0.818
Sm antibodies (positive)	2 (1%)	4 (3.3%)	0.075
RNP antibodies (positive)	2 (1%)	6 (4.9%)	0.015 *
Scl-70 antibodies (positive)	1 (0.5%)	1 (0.8%)	0.748
Jo-1 antibodies (positive)		3 (2.4%)	
Rheumatoid factor ‡	positive: 35 (16.9%)	positive: 22 (17.9%)	<0.001 ***
	>triple: 27 (13%)	>triple: 35 (28.5%)	
Hypergammaglobulinemia (yes)	42 (20.3%)	35 (28.5%)	0.126
ESR ‡ (mm/h)	18 (10–30)	30 (16.25–50)	<0.001 ***
Saxon’s test (positive)	37 (17.9%)	22 (17.9%)	0.619
Saxon’s test difference ‡ (g)	2.65 (1.27–3.90)	3.32 (1.12–3.73)	0.565
Schirmer’s test (positive)	99 (47.8%)	50 (40.7%)	0.607
Schirmer’s test ‡ (lowest value) (mm)	3 (1–5)	3 (1–6)	0.771
Sicca symptoms (yes)	175 (84.5%)	104 (84.6%)	0.788
Systemic involvement (yes)	77 (37.2%)	71 (57.7%)	0.003 **
Hematologic involvement ‡	Lymph node swelling: 9 (4.3%) Hem. disease: 5 (2.4%)	Lymph node swelling: 6 (4.9%) Hem. disease: 2 (1.6%)	0.772
Glandular enlargement (yes)	10 (4.8%)	5 (4.1%)	0.600
Cutane involvement (yes)	49 (23.7%)	49 (39.8%)	0.012 *
Renal involvement (yes)	9 (4.3%)	6 (4.9%)	0.986
Pulmonary involvement (yes)	11 (5.3%)	20 (16.2%)	0.002 **
CVD (yes)	23 (11.9%)	16 (13.7%)	ns
Antihypertensive therapy (yes)	57 (27.5%)	39 (31.7%)	0.218
Statin therapy (yes)	17 (8.2%)	11 (8.9%)	0.723
Cortisone therapy (yes)	45 (21.7%)	65 (52.8%)	<0.001 ***
DMARD (yes)	53 (27.6%)	61 (49.6%)	<0.001 ***
Methotrexate	10 (5.2%)	13 (10.7%)	0.071
Hydroxychloroquine	31 (16.2%)	22 (18.0%)	0.664
Azathioprine	5 (2.6%)	12 (9.8%)	0.006 **
Leflunomide	1 (0.5%)	6 (4.9%)	0.010 *
Rituximab	2 (1.0%)	1 (0.8%)	0.844
TNF inhibitor	1 (0.5%)	6 (4.9%)	0.010 *
Mycofenolat mofetil	1 (0.5%)	2 (1.6%)	0.321
Sulfasalazine	1 (0.5%)	4 (3.3%)	0.057
JAK inhibitors	-	2 (1.6%)	0.321
Further autoimmune diseases (yes)	21 (10.1%)	12 (9.8%)	0.865

‡ Non-normal distribution: presentation as median (interquartile range). † Normal distribution: presentation as mean (S.D.). Others: absolute and relative frequencies. *, **, *** Significant difference between the two groups. Data on glandular area, number of samples, CD3 and CD20 present in 171 FS+/215 FS−, 176 FS+/234 FS−, 185 FS+/237 FS− and 185 FS+/234 FS− patients respectively. HDL: high-density lipoprotein; LDL: low-density lipoprotein; ENA: extractable nuclear antigens; ESR: erythrocyte sedimentation rate; CVD: cardiovascular diseases; DMARD: disease-modifying antirheumatic drugs.

**Table 2:** Descriptive patient characteristics (whole study population).

	Focus Score Positive (*n* = 306)	Focus Score Negative (*n* = 372)	Significance (*p*)
Age † (years)	56.63 ± 12.41	52.93 ± 11.78	<0.001 ***
Gender (female)	275 (91.1%)	335 (90.3%)	0.791
Arterial hypertension (yes)	101 (35.4%)	124 (34.6%)	0.449
Type 2 diabetes (yes)	18 (6.3%)	22 (6.2%)	1
Hyperlipidemia (yes)	77 (42.8%)	134 (54.9%)	0.014 *
Nicotine use (smokers)	47 (19.5%)	103 (34.3%)	<0.001 ***
HDL † (mg/dL)	63.55 ± 17.95	65.79 ± 20.07	0.254
LDL † (mg/dL)	129.96 ± 43.84	133.96 ± 39.30	0.343
Total cholesterol † (mg/dL)	200.78 ± 47.51	207.83 ± 42.09	0.107
Systolic blood pressure † (mmHg)	119.42 ± 16.17	124.57 ± 15.37	0.180
Diastolic blood pressure † (mmHg)	74.18 ± 12.24	77.96 ± 13.47	0.209
Glandular area ‡ (mm^2^)	6.50 (3.70–9.37)	6.42 (3.93–9.93)	0.853
Number of samples ‡	2 (1–2)	2 (1–2)	0.718
CD3 ‡	80 (58–200)	30 (15–60)	<0.001 ***
CD20 ‡	55 (20–150)	8 (1–19.25)	<0.001 ***
ENA screen (positive)	132 (48%)	58 (16.8%)	<0.001 ***
SSA antibodies (positive)	116 (43.4%)	41 (11.8%)	<0.001 ***
SSB antibodies (positive)	37 (13.9%)	12 (3.5%)	<0.001 ***
Rheumatoid factor ‡	positive: 56 (21.7%) >triple: 53 (20.5%)	positive: 31 (9.1%) >triple: 25 (7.3%)	<0.001 ***
Hypergammaglobulinemia (positive)	66 (31.1%)	32 (11.3%)	<0.001 ***
ESR ‡ (mm/h)	20 (12–40)	16 (8–28)	<0.001 ***
CRP ‡ (mg/L)	1.1 (0.3–4.08)	0.84 (0.25–2.45)	0.048 *
ANA ‡ (titer)	80 (0–1120)	0 (0–160)	<0.001 ***
Saxon’s test (positive)	53 (40.8%)	83 (43.9%)	0.645
Saxon’s test difference ‡ (g)	2.34 (0.95–3.68) (*n* = 218)	2.38 (1.34–3.63) (*n* = 284)	0.589
Schirmer’s test (positive)	127 (73.0%)	155 (72.8%)	1
Schirmer’s test ‡ (lowest value (mm))	3 (1–6) (*n* = 161)	3 (2–6) (*n* = 199)	0.358
Sicca symptoms (yes)	250 (91.9%)	294 (85%)	0.009 **
Systemic involvement (yes)	127 (45.5%)	-	-
Hematologic involvement ‡	Lymph node swelling: 12 (4.8%) Hem. Disease: 6 (2.4%)	-	-
Glandular enlargement (yes)	12 (4.9%)	-	-
Glucocorticoids (yes)	97 (34.6%)	122 (34.5%)	1
DMARD (yes)	99 (35.4%)	87 (24.6%)	0.004 **
Further autoimmune diseases (yes)	28 (9.6%)	61 (16.9%)	0.008 **

‡ Non-normal distribution: presentation as median (interquartile range). † Normal distribution: presentation as mean (S.D.). Others: absolute and relative frequencies. *, **, *** Significant difference between the two groups. Data on glandular area, number of samples, CD3 and CD20 present in 171 FS+/215 FS−, 176 FS+/234 FS−, 185 FS+/237 FS− and 185 FS+/234 FS− patients, respectively. HDL: high-density lipoprotein; LDL: low-density lipoprotein; ENA: extractable nuclear antigens; ESR: erythrocyte sedimentation rate; CRP: C-reactive protein; ANA: antinuclear antibodies; DMARD: disease-modifying antirheumatic drugs.

**Table 3:** Descriptive patient characteristics (primary Sjogren’s subgroup (pSS)).

	Focus Score Positive (*n* = 306)	Focus Score Negative (*n* = 372)	Significance (*p*)
Age † (years)	56.45 ± 12.98	54.62 ± 11.49	0.621
Gender (female)	164 (91.1%)	12 (92.3%)	0.884
Arterial hypertension (yes)	58 (33.9%)	7 (53.8%)	0.149
Type 2 diabetes (yes)	9 (5%)	2 (15.4%)	0.142
Hyperlipidemia (yes)	49 (43.8%)	4 (30.8%)	0.374
Nicotine use (smokers)	30 (16.6%)	3 (23.1%)	0.609
HDL † (mg/dL)	62.61 ± 18.15	60.91 ± 20.36	0.770
LDL † (mg/dL)	132.83 ± 46.74	133.73 ± 18.73	0.480
Total cholesterol † (mg/dL)	203.59 ± 49.45	191.15 ± 31.38	0.378
Systolic blood pressure † (mmHg)	119.92 ± 16.66	117.50 ± 12.58	0.777
Diastolic blood pressure † (mmHg)	74.62 ± 13.58	72.50 ± 28.72	0.783
Gland area ‡ (mm^2^)	7.17 (3.80–10.66)	6.11 (3.92–9.19)	0.513
Number of samples ‡	2 (1.25–2)	2 (1.25–2)	0.587
CD3 ‡	80 (59.75–185)	25.50 (15.75–39.25)	<0.001 ***
CD20 ‡	57.50 (20.75–121.50)	10.5 (1.75–24.75)	<0.001 ***
ENA screen (positive)	75 (45.5%)	5 (38.5%)	0.628
SSA antibodies (positive)	65 (40.9%)	5 (38.5%)	0.865
SSB antibodies (positive)	22 (13.8%)	0 (0%)	0.153
Rheumatoid factor ‡	positive: 32 (20.5%)		
>triple: 22 (14.1%)	positive: 1 (7.7%)		
>triple: 0 (0%)	0.047 *		
Hypergammaglobulinemia (yes)	35 (27.3%)	2 (18.2%)	0.513
ESR ‡ (mm/h)	18 (10–30)	15 (14–22)	0.827
Saxon’s test (positive)	32 (39.5%)	2 (16.7%)	0.128
Saxon’s test difference ‡ (g)	2.64 (1.19–3.85) (*n* = 132)	3.2 (2.8–4.43) (*n* = 11)	0.090
Schirmer’s test (positive)	85 (75.2%)	8 (88.9%)	0.606
Schirmer’s test ‡ (lowest value) (mm)	3 (1–5) (*n* = 106)	3 (1–4) (*n* = 9)	0.615
Sicca symptoms (yes)	152 (91.6%)	11 (91.7%)	0.988
Systemic involvement (yes)	67 (40.4%)	4 (30.8%)	0.499
Hematologic involvement ‡	Lymph node swelling: 6 (4.2%) Hem. Disease: 4 (2.8%)	Lymph node swelling: 1 (7.7%) Hem. Disease: 0 (0%)	0.847
Glandular enlargement (yes)	8 (5.6%)	1 (7.7%)	0.758
Cortisone therapy (yes)	38 (22.9%)	3 (23.1%)	0.988
DMARD (yes)	45 (26.9%)	5 (38.5%)	0.375
Further autoimmune diseases (yes)	17 (9.6%)	2 (15.4%)	0.505

‡ Non-normal distribution: presentation as median (interquartile range). † Normal distribution: presentation as mean (S.D.). Others: absolute and relative frequencies. *, *** Significant difference between the two groups. Data on glandular area, number of samples, CD3 and CD20 present in 100 FS+/12 FS−, 108 FS+/12 FS−, 110 FS+/12 FS− and 110 FS+/12 FS− patients, respectively. HDL: high-density lipoprotein; LDL: low-density lipoprotein; ENA: extractable nuclear antigens; ESR: erythrocyte sedimentation rate; DMARD: disease-modifying antirheumatic drugs.

**Table 4:** Diagnostic value of SSA and SSB antibodies, ENA screening, sicca, Schirmer’s test and Saxon’s test compared to MLSG biopsy findings (reference: focus score ≥ ¼ mm^2^).

	Specificity (%)	Sensitivity (%)	Area under the Curve (95%-CI)	Significance (*p*)
SSA-Ro	88.2	43.4	0.658 (0.613, 0.703)	<0.001 ***
SSB-La	96.5	13.9	0.552 (0.506, 0.599)	0.027 *
Any ENA	66.8	69.5	0.656 (0.612, 0.700)	<0.001 ***
Sicca symptoms	15	91.9	0.535 (0.489, 0.580)	0.031 *
Schirmer’s test	27.3	73	0.507 (0.450, 0.565)	0.574
Saxon’s test	57.9	39	0.516 (0.451, 0.580)	0.633

*, *** Significant differences between the two groups.

**Table 5:** Associations of focus score with immunohistochemical markers, clinical and laboratory characteristics (whole population and primary Sjogren’s syndrome (pSS)).

	Focus Score (Whole Population)		Focus Score (pSS)	
	rho or Median (IQR)	*p*	rho or Mean (IQR)	*p*
Age † (years)	0.161	<0.001 ***	0.110	0.132
Gender Female Male	1 (0–2) 0.73 (0–1.6)	0.274	1.8 (1–3) 1.46 (1.14–3)	0.614
Arterial hypertension Yes No	0.79 (0–1.82) 1 (0–2.09)	0.162	1.8 (1–3.5) 1.44 (1–3)	0.492
Type 2 diabetes Yes No	1.03 (0–2.56) 1 (0–2)	0.493	2.1 (1.28–4.3) 1.6 (1–3)	0.234
Hyperlipidemia Yes No	0.37 (0–1.4) 0.82 (0–2)	0.044 *	1.87 (1.1–2.92) 1.74 (1.04–3.38)	0.881
Nicotine use Yes Ex No	0.42 (0–1.05) 1 (0–2.38) 1 (0–2.1)	0.002 **	1.28 (1–2.78) 1.82 (1.17–4) 1.99 (1.06–3)	0.146
HDL † (mg/dL)	−0.046	0.363	−0.091	0.334
LDL † (mg/dL)	−0.076	0.132	0.000	0.998
Total cholesterol † (mg/dL)	−0.117	0.016 *	−0.057	0.526
Systolic blood pressure † (mmHg)	−0.073	0.477	0.048	0.728
Diastolic blood pressure † (mmHg)	−0.269	0.007 **	−0.164	0.226
Gland area ‡ (mm^2^)	0.025	0.620	−0.267	0.004 **
Number of samples ‡	0.073	0.142	−0.058	0.529
CD3 ‡	0.659	<0.001 ***	0.431	<0.001 ***
CD20 ‡	0.692	<0.001 ***	0.387	<0.001 ***
ENA Screen Positive Negative	1.55 (1–3.05) 0.47 (0–1.42)	<0.001 ***	2.1 (1–4) 1.43 (1–2.23)	0.028 *
SSA Antibodies Positive Negative	2 (1–3.36) 0.49 (0–1.39)	<0.001 ***	2.21 (1–4) 1.41 (1–2.21)	0.010 *
SSB Antibodies Positive Negative	1.81 (1–3.66) 0.95 (0–1.99)	<0.001 ***	2.65 (1–5.25) 1.48 (1–2.99)	0.099
Rheumatoid factor Positive Negative	1.51 (0.47–3.09) 0.63 (0–1.31)	<0.001 ***	2.99 (1.44–4.13) 1.29 (1–2.22)	<0.001 ***
Hypergammaglobulinemia Positive Negative	1.27 (0.71–3.36) 0.73 (0–1.76)	<0.001 ***	2.3 (1.09–4) 1.44 (1–2.61)	0.024 *
ESR ‡ (mm/h)	0.235	<0.001 ***	0.302	<0.001 ***
CRP ‡ (mg/L)	−0.023	0.600	0.178	0.019 *
ANA ‡ (titer)	0.280	<0.001 ***	0.312	<0.001 ***
Saxon’s test Positive Negative	0.55 (0–1.54) 0.54 (0–1.46)	0.785	1.83 (1.04–2.81) 1.4 (1–2.76)	0.235
Saxon’s difference ‡ (g)	−0.068	0.171	−0.135	0.109
Schirmer’s test Positive Negative	0.78 (0–1.93) 0.8 (0–1.72)	0.585	1.7 (1–3.44) 1.3 (1–2.08)	0.147
Schirmer’s test ‡ (lowest value (mm))	−0.065	0.234	−0.162	0.085
Sicca symptoms Yes No	1 (0–2.01) 0.21 (0–1.4)	0.006 *	1.82 (1–3) 1.66 (1–3)	0.924
Systemic involvement Yes No	-	-	2 (1.1–4) 1.44 (1–2.62)	0.006 *
Hematologic involvement ‡ Lymph node swelling Hematologic disease No manifestations	-	-	4 (1–5) 5.03 (1.72–9.3) 1.69 (1–3)	0.002 **
Glandular enlargement Yes No	-	-	4 (2.98–6.2) 1.6 (1–3)	0.007 *
Cortisone therapy Yes No	1 (0–2.47) 1 (0–1.86)	0.255	2.01 (1–3.75) 1.53 (1–2.99)	0.353
DMARD Yes No	1 (0–2.34) 0.87 (0–1.8)	0.004 **	2 (1–3.03) 1.46 (1–3)	0.806
Further autoimmune diseases Yes No	0 (0–1.64) 1 (0–2)	0.010 *	1.96 (1.28–2.75) 1.7 (1–3)	0.510

‡ Non-normal distribution. † Normal distribution. Quantitative characteristics: Spearman’s test (non-normal distribution; rho). Qualitative characteristics: Mann–Whitney U-test, ANOVA. *, **, *** Significant difference between the two groups. IQR: interquartile rate; HDL: high-density lipoprotein; LDL: low-density lipoprotein; ENA: extractable nuclear antigens; ESR: erythrocyte sedimentation rate; CRP: C-reactive protein; ANA: antinuclear antibodies.

**Table 6:** Associations of immunohistochemical markers with clinical and laboratory patient characteristics.

	CD3		CD20		CD21		IgG4	
	rho or Median (IQR)	*p*	rho or Mean (IQR)	*p*	rho or Mean (IQR)	*p*	rho or Mean (IQR)	*p*
Age † (years)	0.125	0.010 *	0.119	0.015 *	0.119	0.015 *	0.041	0.397
Gender Female Male	56 (25–100) 50 (22–75)	0.606	17 (4–55) 17 (3–53)	0.734	0 (0–0) 0 (0–0)	0.717	0 (0–0) 0 (0–0)	0.598
Arterial hypertension Yes No	54.5 (30–100) 56 (20–100)	0.327	17 (5–52) 17 (4–58.75)	0.722	0 (0–0) 0 (0–0)	0.584	0 (0–0) 0 (0–0)	0.836
Type 2 diabetes Yes No	59.5 (50–135) 54 (23–100)	0.095	30 (7.5–90) 15 (4–53)	0.082	0 (0–0) 0 (0–0)	0.003 **	0 (0–0) 0 (0–0)	0.348
Hyperlipidemia Yes No	50 (20–100) 58 (30–120)	0.062	12 (4–50) 20 (5–70)	0.016 *	0 (0–0) 0 (0–0)	0.229	0 (0–0) 0 (0–0)	0.215
Nicotine use Yes No/Ex	50 (20–74.5) 59 (27–105)	0.022 *	10 (1–23) 20 (5–64)	<0.001 ***	0 (0–0) 0 (0–0)	0.107	0 (0–0) 0 (0–0)	0.370
HDL † (mg/dL)	−0.058	0.271	−0.059	0.268	−0.045	0.396	−0.091	0.082
LDL † (mg/dL)	−0.136	0.010 *	−0.140	0.008 **	−0.045	0.403	−0.069	0.183
Total cholesterol † (mg/dL)	−0.174	0.001 **	−0.172	0.001 **	−0.080	0.120	−0.099	0.047 *
Systolic blood pressure † (mmHg)	−0.028	0.818	−0.076	0.524	0.125	0.303	−0.139	0.227
Diastolic blood pressure † (mmHg)	−0.180	0.130	−0.293	0.012 *	−0.049	0.684	−0.227	0.047 *
Gland area ‡ (mm^2^)	0.228	<0.001 ***	0.208	<0.001 ***	0.038	0.461	0.051	0.314
Number of samples ‡	0.144	0.005 **	0.115	0.025 *	0.048	0.357	0.057	0.261
Focus Score	0.659	<0.001 ***	0.692	<0.001 ***	0.292	<0.001 ***	0.118	0.014 *
ENA Screen Positive Negative	60 (31.5–120) 50 (20–100)	0.030 *	37 (11–91.5) 12 (3–50)	<0.001 ***	0 (0–0) 0 (0–0)	0.061	0 (0–0) 0 (0–0)	0.195
SSA Antibodies Positive Negative	69 (40–195) 50 (20–100)	0.002 **	50 (14–112) 12 (3–48)	<0.001 ***	0 (0–0) 0 (0–0)	0.009 **	0 (0–0) 0 (0–0)	0.187
SSB Antibodies Positive Negative	100 (44–375) 53 (23.25–100)	0.004 **	40 (11–192.5) 15 (4–51)	0.039 *	0 (0–0) 0 (0–0)	0.003 **	0 (0–0) 0 (0–0)	0.027 *
Rheumatoid factor Positive Negative	67.5 (50–150) 50 (20–80)	<0.001 ***	42 (10–131) 12 (2.5–40.5)	<0.001 ***	0 (0–0) 0 (0–0)	0.047 *	0 (0–0) 0 (0–0)	0.147
Hypergammaglobulinemia Positive Negative	70 (43–135) 60 (20–100)	0.009 **	50 (14.25–111.5) 12 (2–50)	<0.001 ***	0 (0–0) 0 (0–0)	0.586	0 (0–0) 0 (0–0)	0.030 *
ESR ‡ (mm/h)	0.195	<0.001 ***	0.233	<0.001 ***	0.122	0.019 *	0.128	0.011 *
CRP ‡ (mg/L)	0.052	0.307	0.108	0.035 *	0.049	0.345	0.141	0.005 **
ANA ‡ (titer)	0.109	0.038 *	0.229	<0.001 ***	0.018	0.741	0.065	0.212
Saxon’s test Positive Negative	50 (25.5–100) 50 (20–84.75)	0.548	18.5 (5–50) 12 (4.25–55)	0.757	0 (0–0) 0 (0–0)	0.990	0 (0–0) 0 (0–0)	0.558
Saxon’s difference ‡ (g)	−0.068	0.228	−0.021	0.715	−0.008	0.888	−0.044	0.433
Schirmer’s test Positive Negative	57 (27–100) 56 (20–100)	0.963	17 (5–70) 16.5 (5–50)	0.582	0 (0–0) 0 (0–0)	0.080	0 (0–0) 0 (0–0)	0.113
Schirmer’s test ‡ (lowest value (mm))	0.046	0.430	−0.008	0.896	−0.096	0.104	−0.060	0.295
Sicca symptoms Yes No	57 (26.5–100) 50 (20–63.5)	0.199	17 (4.25–55) 18 (2–50)	0.479	0 (0–0) 0 (0–0)	0.276	0 (0–0) 0 (0–0)	0.704
Systemic involvement Yes No	57.5 (20–100) 54 (27–100)	0.956	14 (2–73.75) 19 (5–50)	0.807	0 (0–0) 0 (0–0)	0.060	0 (0–0) 0 (0–0)	0.720
Hematologic involvement ‡ Lymph node swelling Hematologic disease No manifestations	53.5 (13.5–115) 95 (35–127.5) 55 (26–100)	0.947	10 (1–57.5) 40 (2.5–322.5) 17 (5–52)	0.033 *	0 (0–0) 0 (0–0) 0 (0–0)	0.978	0 (0–0) 0 (0–0) 0 (0–0)	0.980
Glandular enlargement Yes No	100 (60–287.5) 55 (23–100)	0.025 *	100 (10–300) 16.5 (4–52.75)	0.091	0 (0–0) 0 (0–0)	0.296	0 (0–0) 0 (0–0)	0.821
Cortisone therapy Yes No	56.5 (26.25–115) 55 (24–100)	0.542	20 (3–61.75) 15 (4.4–50)	0.396	0 (0–0) 0 (0–0)	0.352	0 (0–0) 0 (0–0)	0.111
DMARD Yes No	57 (30–87) 54 (23–100)	0.710	20 (3–55) 15.5 (4.8–51.3)	0.704	0 (0–0) 0 (0–0)	0.272	0 (0–0) 0 (0–0)	0.871
Further autoimmune diseases Yes No	50 (20–100) 57 (25–100)	0.368	10 (3–42) 19.5 (4.55)	0.199	0 (0–0) 0 (0–0)	0.168	0 (0–0) 0 (0–0)	0.192

‡ Non-normal distribution. † Normal distribution. Quantitative characteristics: Spearman’s test (non-normal distribution; rho). Qualitative characteristics: Mann–Whitney U-test, ANOVA. *, **, *** Significant difference between the two groups.

**Table 7:** Fibromyalgia at admission to and release from the hospital in patients with primary, secondary and without SS and in terms of positive and negative focus score.

	Primary SS	Secondary SS	No SS	Significance (*p*)
FM at admission to the hospital (yes)	50 (31.4%)	17 (10.7%)	92 (57.9%)	0.021 *
FM at release from the hospital (yes)	101 (29.4%)	38 (11.0%)	205 (59.6%)	<0.001 ***
	**Focus Score positive**		**Focus Score negative**	**Significance (*p*)**
FM at admission to the hospital (yes)	63 (39.6%)		96 (60.4%)	0.124
FM at release from the hospital (yes)	127 (37.8%)		209 (62.2%)	0.001 ***

*, *** Significant differences between the two groups. FM: fibromyalgia; SS: Sjogren’s syndrome.

## Data Availability

Data are available upon reasonable request by any qualified researchers who engage in rigorous, independent scientific research, and will be provided following review and approval of a research proposal and Statistical Analysis Plan (SAP) and execution of a Data Sharing Agreement (DSA). All data relevant to the study are included in the article.
